# Siamese Crocodile (*Crocodylus siamensis*) Meat Protein Hydrolysate Enhances Antioxidant Activity and Attenuates Airway Lesions in Ovalbumin-Induced Asthmatic Rats

**DOI:** 10.3390/foods15213885

**Published:** 2026-10-30

**Authors:** Phitsanu Tulayakul, Payu Srisuporn, Rungrueang Yodsheewan, Wasaporn Preteseille Chanput, Urai Pongchairerk, Jirakul Jithan, Supinya Samart, Wirasak Fungfuang

**Affiliations:** 1 Department of Veterinary Public Health, Faculty of Veterinary Medicine, Kasetsart University, Kamphaeng Saen Campus, Nakhon Pathom 73140, Thailand; fvetpnt@ku.ac.th; 2 Department of Companion Animal Clinical Sciences, Faculty of Veterinary Medicine, Kasetsart University, Bangkok 10900, Thailand; payu.s@ku.th; 3 Department of Pathology, Faculty of Veterinary Medicine, Kasetsart University, Kamphaeng Saen Campus, Nakhon Pathom 73140, Thailand; rungrueang.y@ku.th; 4 Department of Food Science and Technology, Faculty of Agro-Industry, Kasetsart University, Bangkok 10900, Thailand; wasaporn.c@gmail.com; 5 Department of Veterinary Anatomy, Faculty of Veterinary Medicine, Kasetsart University, Bangkok 10900, Thailand; fveturp@ku.ac.th; 6 Department of Zoology, Faculty of Science, Kasetsart University, Bangkok 10900, Thailand; jirakul1102@gmail.com (J.J.); supinya230546@gmail.com (S.S.)

**Keywords:** allergic asthma, crocodile, hydrolyzed, antioxidant, rat

## Abstract

Allergic asthma involves airway inflammation, oxidative imbalance, and structural injury. This study evaluated the effects of Siamese crocodile (*Crocodylus siamensis*) meat protein hydrolysate on serum antioxidant enzyme activities, hematological and biochemical parameters, and airway histopathology in an ovalbumin-induced rat model of allergic asthma. Forty-two Sprague-Dawley rats were allocated to six groups: healthy control, untreated asthma, and asthma treated with crocodile meat protein hydrolysate (100 or 200 mg/kg), commercial chicken essence (100 mg/kg), and dexamethasone (2.5 mg/kg). Treatments were administered for 14 days. Food intake, body weight, hematological indices, serum biochemical parameters, and superoxide dismutase (SOD) and catalase (CAT) activities were measured, and tracheal and pulmonary histopathological findings were evaluated qualitatively, without inferential statistical comparison. Food intake did not differ significantly among groups. Neither hydrolysate dose significantly altered body weight or most measured hematological and biochemical parameters relative to the healthy control or untreated asthma group. Dexamethasone was associated with lower body weight and platelet counts, neutrophilia, lymphopenia, increased alanine and aspartate aminotransferase activities, and altered serum protein and lipid profiles. SOD activity was higher in both hydrolysate-treated asthma groups than in the untreated asthma group, whereas CAT was higher in the hydrolysate-treated asthma groups than in the dexamethasone group. Existing histopathological descriptions and representative sections suggested less pronounced epithelial injury, inflammatory, infiltration, hemorrhage and vascular changes in the hydrolysate groups than in untreated asthma. These qualitative observations did not establish statistically significant histological differences or dose dependence. Hydrolysate administration was associated with higher serum SOD activity than untreated asthma, while qualitative histopathological observations suggested less pronounced airway lesions. These findings support further preclinical investigation of the hydrolysate as a potential food-derived bioactive ingredient. However, they do not confirm efficacy against asthma, establish that antioxidant responses mediated the histopathological observations, or demonstrate long-term safety.

## 1. Introduction

Asthma is among the most prevalent chronic inflammatory diseases of the respiratory tract, affecting an estimated 300–400 million individuals globally, with an incidence that continues to rise particularly in developing countries [1]. Its hallmark pathophysiology of asthma involves airway hyperresponsiveness, mast cell degranulation, eosinophilic infiltration, goblet cell metaplasia, and subepithelial fibrosis driven by a T-helper 2 (Th2)-skewed immune response alongside the upregulation of pro-inflammatory cytokines including interleukin (IL)-4, IL-5, and IL-13, and immunoglobulin E (IgE) [2]. Concurrently, oxidative stress amplifies airway inflammation and tissue damage, with reactive oxygen species contributing to epithelial injury and perpetuating the inflammatory cascade. These processes lead to progressive structural remodeling of the airways, manifesting clinically as recurrent wheezing, dyspnea, cough, and variable airflow obstruction. Oxidative imbalance can accompany inflammatory processes and epithelial injury. SOD converts superoxide to hydrogen peroxide, whereas CAT contributes to hydrogen peroxide decomposition. Their activities provide complementary information on enzymatic antioxidant defenses. Serum measurements, however, do not directly quantify oxidative damage or antioxidant activity within the lung. Current first-line pharmacological management relies on inhaled corticosteroids and bronchodilators; however, long-term or high-dose systemic corticosteroid exposure carries substantial risks including hypothalamic–pituitary–adrenal (HPA) axis suppression, hepatocellular injury, hyperlipidemia, muscle wasting, osteoporosis, and secondary immunosuppression [3]. Inhaled corticosteroids are a cornerstone of asthma management. Adverse effects associated with prolonging of high-dose systemic corticosteroid exposure should be distinguished from the clinical role of inhaled therapy. The investigation of food-derived bioactive ingredients is therefore a preclinical research strategy and does not imply replacement of established asthma treatment.

The Siamese crocodile (*Crocodylus siamensis*) is a critically endangered freshwater reptile natively in Southeast Asia that it is widely farmed commercially in Thailand. Beyond its economic value as a source of leather and meat, crocodile-derived products have been used in traditional medicine across Asian cultures for purposes of their health-promoting and immunomodulatory effects [4]. Previous investigation of crocodile oil and liver extracts have examined metabolic and biotransformation-related endpoints [5,6,7,8,9,10,11], but these products differ from meat protein hydrolysates. Direct studies of crocodile meat-derived peptides have reported antioxidant activity using different preparation and testing approaches [12,13]. Studies of blood components and hemoglobin hydrolysates provide additional, indirect evidence of crocodile-derived bioactives [14,15]. The meat of *C. siamensis* is a rich source of high-quality protein, and its enzymatic hydrolysis yields bioactive peptide fragments with potent anti-inflammatory and antioxidant properties. Despite anecdotal and ethnopharmacological evidence suggesting that crocodile-derived products may benefit patients with respiratory conditions, no rigorous in vivo study has systematically evaluated the safety and efficacy of crocodile meat peptide extracts in a validated asthma model.

In the present study, two doses of crocodile meat hydrolysate (100 and 200 mg/kg) were evaluated relative to an untreated asthma control, using an established ovalbumin (OVA)-induced model of allergic asthma in Sprague-Dawley rats [16]. A commercial chicken essence supplement (100 mg/kg) was included as an animal-derived food comparator and dexamethasone (2.5 mg/kg) was used as a pharmacological comparator. The food comparison does not isolate species-specific peptide activity without compositional matching. We hypothesized that hydrolysate administration would be associated with higher serum antioxidant enzyme activities and less pronounced airway histopathological injury. The objective was to assess these endpoints together with body weight, food intake, and hematological and biochemical parameters. These findings could provide a feasible strategy for the high value of Siamese crocodile meat as a potential food-derived ingredient.

## 2. Materials and Methods

### 2.1. Preparation of Crocodile Meat Protein Hydrolysate

The crocodile meat was acquired from a licensed commercial facility operating under regulatory compliance. The meat containing 20.11% protein (AOAC 2019, [17]) was sliced and minced, mixed with distilled water at a 1:1 (*w*/*w*) ratio in a double-jacketed steam mixer, and hydrolyzed with 3% (*w*/*w* enzyme-to-substrate ratio) Amano 2SD (Amano Enzyme Inc.Nagoya, Japan) for 3 h. The reaction was maintained at 50 °C at neutral pH). After hydrolysis, the mixture underwent phase separation using a pilot-scale basket centrifuge. The resulting light-yellow liquid hydrolysate was collected and stored at −20 °C until analysis.

The final protein content of the hydrolysate was 10.29 g/100 mL (AOAC 2019), corresponding to a degree of hydrolysis of 76.28%. Visualization via SDS-PAGE indicated that the molecular weight of the protein hydrolysate ranged between 10 and 15 kDa.

### 2.2. Animals and Experimental Design

Seven-week-old male Sprague-Dawley (SD) rats (200–250 g, *n* = 42) were purchased from Nomura Siam International Co., Ltd. (Bangkok, Thailand). The animals were housed in polycarbonate cages under a 12 h/12 h light/dark cycle at a controlled ambient temperature of 24 ± 2 °C and with 55–60% relative humidity. The rats had free access to standard rat chow and purified water. All experimental procedures complied with institutional ethical guidelines for the care and use of laboratory animals and were approved by the Institutional Animal Care and Use Committee (IACUC) of Kasetsart University, Thailand (Approval No. ACKU66-VET-062; approved on 6 June 2023). The study included only male Sprague-Dawley rats aged seven weeks at study entry, with seven animals allocated to each experimental group. Restriction to a single sex and age provided a more homogeneous experimental population; however, the findings cannot be assumed to apply to female rats or other age groups.

Rats were randomly divided into six groups (*n* = 7 per group): normal control (C group); OVA-induced asthma control (A group); OVA-induced asthma treated with crocodile meat protein hydrolysate at 100 mg/kg (AC100 group); OVA-induced asthma treated with crocodile meat protein hydrolysate at 200 mg/kg (AC200 group); OVA-induced asthma treated with commercial chicken essence at 100 mg/kg (AB group) and OVA-induced asthma treated with dexamethasone at 2.5 mg/kg (AD group). Asthma was induced based on previously established protocol [18]. Briefly, rats in all groups except the C group were sensitized via the intraperitoneal injection of 1 mg of OVA emulsified with 200 mg of aluminum hydroxide in 1 mL of sterile 0.9%NaCl on day 0 and day 7. From day 14 to 28, the animals were challenged daily with aerosolized 1% OVA solution via nebulization for 30 min. Crocodile meat protein hydrolysate or chicken essence were administered intragastrically once daily for 14 consecutive days, 30 min prior to nebulization. Measured outcomes included body weight, food intake, hematological indices, serum hepatic and renal biochemical parameters, lipid profiles, and serum superoxide dismutase and catalase activities. Tracheal and pulmonary histopathological findings were assessed qualitatively.

On day 29, all animals were euthanized via the injection of Tiletamine-Zolazepam (40 mg/kg; intraperitoneal). Whole blood samples were collected for hematological analysis, and serum was isolated and stored at –20 °C for biochemical assays. Tracheal and pulmonary tissues were harvested for histopathological evaluation.

### 2.3. Hematological and Serum Biochemical Analysis

Complete blood count parameters including white blood cell count, differential leukocyte counts (neutrophils, lymphocytes, monocytes, eosinophils, and basophils), red blood cell count, hemoglobin concentration, hematocrit, and platelet count were determined using an automated hematology analyzer. Blood smears were prepared and stained with Wright-Giemsa for morphological evaluation.

Serum was separated by centrifugation at 3000 rpm for 15 min at 4 °C. Hepatic function markers, including alanine aminotransferase (ALT), aspartate aminotransferase (AST), alkaline phosphatase (ALP), albumin, and total protein, were measured using an automated biochemical analyzer. Renal function was assessed by determining serum creatinine and blood urea nitrogen (BUN) levels. Lipid profile, including total cholesterol, high-density lipoprotein cholesterol (HDL-C), and low-density lipoprotein cholesterol (LDL-C), was determined using enzymatic colorimetric assays [19,20]. The hematological parameters were determined using an automatic analyzer (7080 series; Hitachi, Tokyo, Japan).

### 2.4. Antioxidant Activity Assessment

Total protein concentration in serum samples was determined using the Bradford assay, with bovine serum albumin as the calibration standard. Serum catalase (CAT) and superoxide dismutase (SOD) activities were measured spectrophotometrically, normalized to total serum protein content, and expressed as units per milligram of protein (U/mg protein).

CAT activity was measured according to the method described by Mokhtari-Zaer et al. [21], with minor modifications based on the CAT-mediated decomposition of hydrogen peroxide. The reaction mixture contained 1.95 mL of 50 mM potassium phosphate buffer (pH 7.0), 1.00 mL of 10 mM H_2_O_2_, and 0.05 mL of serum, yielding a final volume of 3 mL. The reaction was initiated by adding hydrogen peroxide, and the decrease in absorbance at 240 nm was continuously recorded for 3 min using a spectrophotometer.

SOD activity was measured according to the method described by Radovanović et al. [22], with minor modifications based on the SOD-mediated inhibition of epinephrine autoxidation to adrenochrome. Adrenochrome formation was monitored spectrophotometrically by measuring the increase in absorbance at 480 nm at 26 °C. One unit of SOD activity was defined as the amount of enzyme required to inhibit epinephrine autoxidation by 50% under the specified assay conditions.

### 2.5. Histopathological Analysis

Following euthanasia, tracheal and pulmonary tissues were collected and fixed in 10% neutral-buffered formalin. Tissues were routinely processed, embedded in paraffin, sectioned at 4 μm, and stained with hematoxylin and eosin (H&E). Histopathological examination was performed by a board-certified veterinary pathologist blinded to experimental group allocation. Tracheal examination addressed epithelial desquamation, ciliary loss, submucosal inflammatory cell infiltration, and luminal hemorrhage. Pulmonary examination addressed peribronchial and perivascular inflammatory cell infiltration, alveolar architectural changes, alveolar hemorrhage, vascular congestion, and edema.

The findings are reported qualitatively using the existing pathological descriptions and representative photomicrographs. The available records did not permit verification of the number of sections or microscopic fields examined per animal or reconstruction of an animal-level lesion score. No additional slide examination was undertaken during revision. Consequently, numerical lesion grades are not reported, and the descriptive findings are not used to estimate lesion frequency, between-animal variability, or statistically significant treatment effects.

### 2.6. Statistical Analysis

Quantitative outcomes were summarized as mean ± standard deviation. Distributional normality was assessed using the Shapiro–Wilk test. For outcomes analyzed using one-way ANOVA, pairwise group comparisons were performed using Tukey’s multiple-comparisons procedure. For outcomes analyzed using the Kruskal–Wallis test, Dunn’s multiple-comparisons procedure was used. A *p*-value below 0.05 was considered statistically significant. These multiple-comparisons procedures addressed comparisons among groups within each endpoint and did not, by themselves, control multiplicity across the full set of measured outcomes. All statistical analyses and graphical presentations were performed using GraphPad Prism version 8.0 (GraphPad Software, San Diego, CA, USA). Histopathological findings were presented descriptively, without inferential statistical analysis. Animal-level ordinal scores and quantitative morphometric measurements were unavailable for analysis. The statistical procedures used for quantitative hematological and biochemical outcomes were therefore not applied to histopathology.

## 3. Results

### 3.1. Subsection Effect of Crocodile Meat Hydrolysate on Body Weight and Food Intake

**Figure 1:**
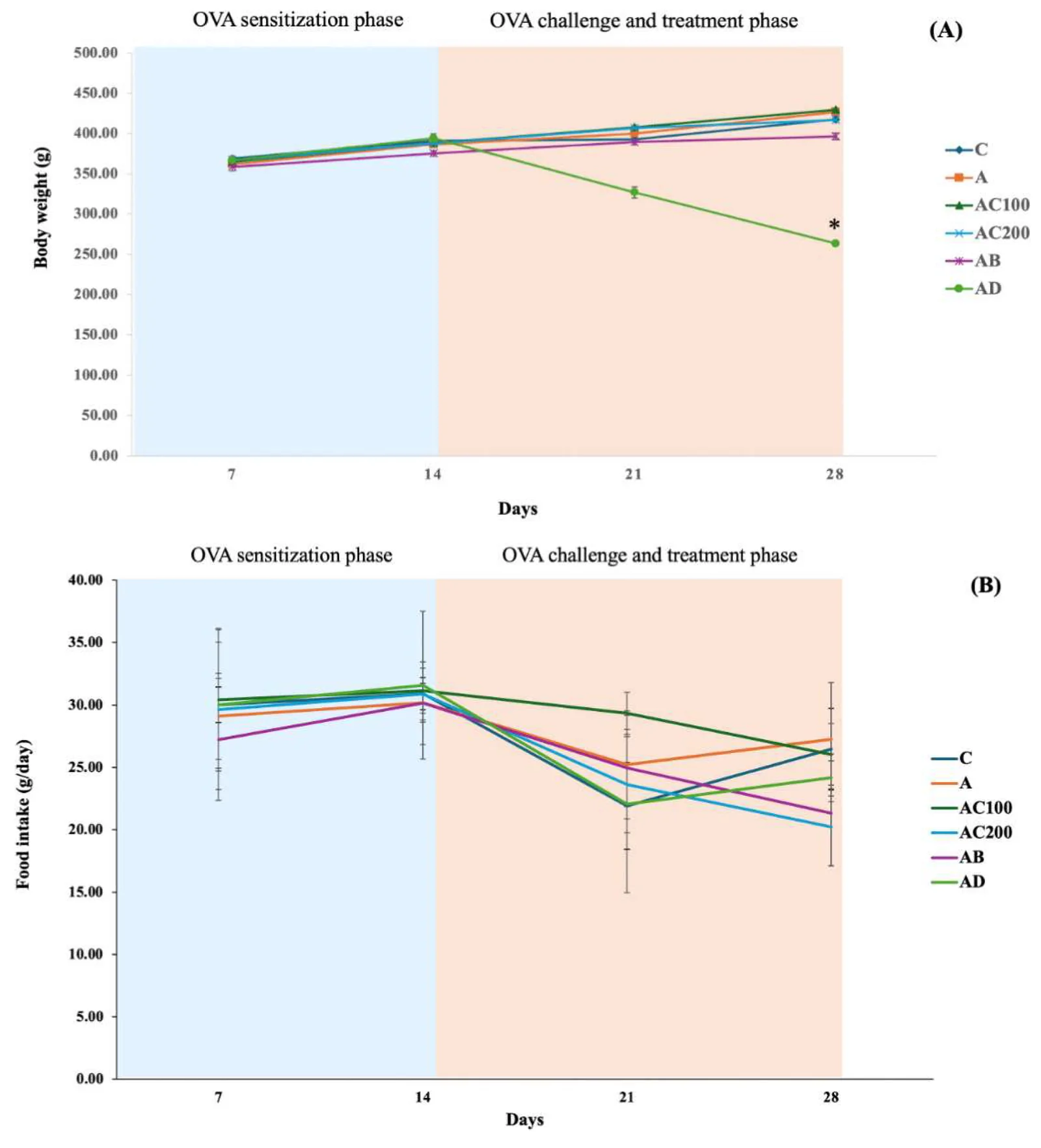
The effect of crocodile meat protein hydrolysate on (**A**) body weight and (**B**) food intake in OVA-induced asthmatic rats. Values are expressed as means ± SD. * *p* < 0.001.

Body weight and food intake throughout the experimental period are shown in Figure 1. During the OVA sensitization phase (days 1–14), body weight increased similarly across all groups. During the OVA challenge and treatment phase (days 15–28), body weight continued to increase in the control, untreated asthma, AC100, AC200, and chicken essence groups. In contrast, body weight decreased progressively in the dexamethasone-treated group and was significantly lower than in all other groups on day 28 (268.4 ± 20.9 g; *p* < 0.001; Figure 1A). Body weight did not differ significantly among the control, untreated asthma, AC100, AC200, and chicken essence groups at the end of the experiment (*p* > 0.05).

Food intake was comparable among groups during the OVA sensitization phase and increased slightly between days 7 and 14 (Figure 1B). Food intake decreased in most groups following OVA challenge on day 21, with partial recovery in several groups by day 28. No significant overall treatment effect or treatment-by-time interaction was detected (*p* > 0.05). Thus, administration of crocodile meat protein hydrolysate at either dose did not significantly affect body weight or food intake relative to the control or untreated asthma group.

### 3.2. Effects of Crocodile Meat Protein Hydrolysate on Hematological and Serum Biochemical Parameters

Hematological parameters are summarized in Table 1. Total white blood cell counts did not differ significantly among the groups (*p* > 0.05). The AD group exhibited a significantly higher neutrophil percentage (83.7% ± 2.5%) and a lower lymphocyte percentage (8.7% ± 2.4%) than all the other groups (*p* < 0.001). Red blood cell counts, hemoglobin concentration, hematocrit, and monocyte, eosinophil, and basophil percentages did not differ significantly among the groups (*p* > 0.05). Platelet counts were significantly lower in the AD group (617.8 ± 184.7 × 10^3^/µL) than in all the other groups (*p* < 0.05), although the mean remained within the reported reference range. In asthmatic rats, neither dose of crocodile meat protein hydrolysate significantly altered the measured hematological parameters relative to the control or untreated asthma group.

**Figure 2:**
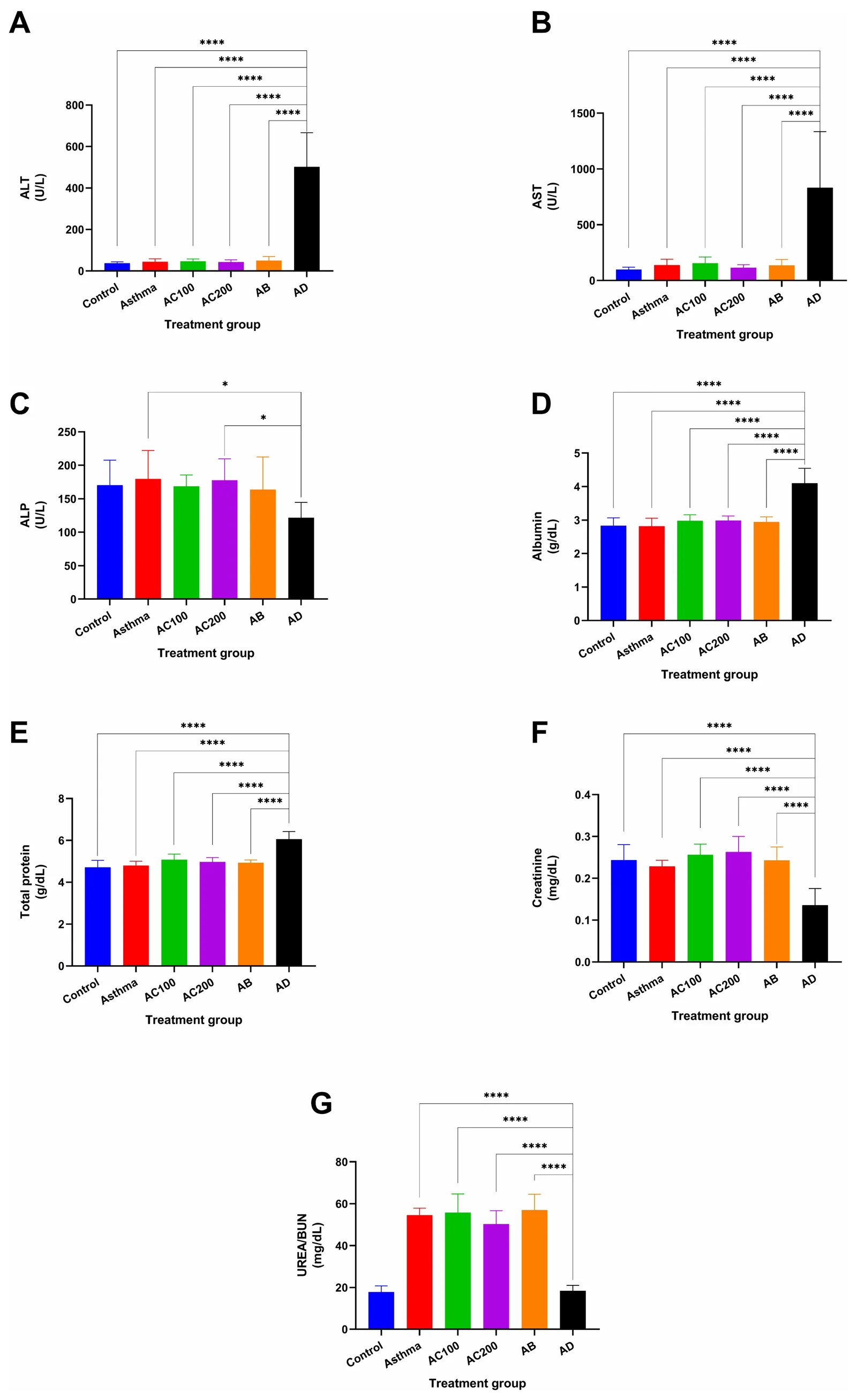
Serum hepatic and renal function parameters across treatment groups. (**A**) ALT, (**B**) AST, (**C**) ALP, (**D**) Albumin, (**E**) Total Protein, (**F**) Creatinine and (**G**) UREA/BUN. The AD group showed significantly elevated ALT, AST, Albumin, and Total Protein (*p* < 0.0001), and significantly reduced ALP and Creatinine (*p* < 0.05), compared to all other groups. For UREA/BUN (**G**), the Control and AD groups did not differ significantly, whereas both showed significantly lower levels than the Asthma, AC100, AC200, and AB groups (all *p* < 0.0001). Values are expressed as mean ± SD. Statistical analysis was performed using one-way ANOVA followed by Tukey’s multiple comparisons test. * *p* < 0.05; **** *p* < 0.0001.

**Figure 3:**
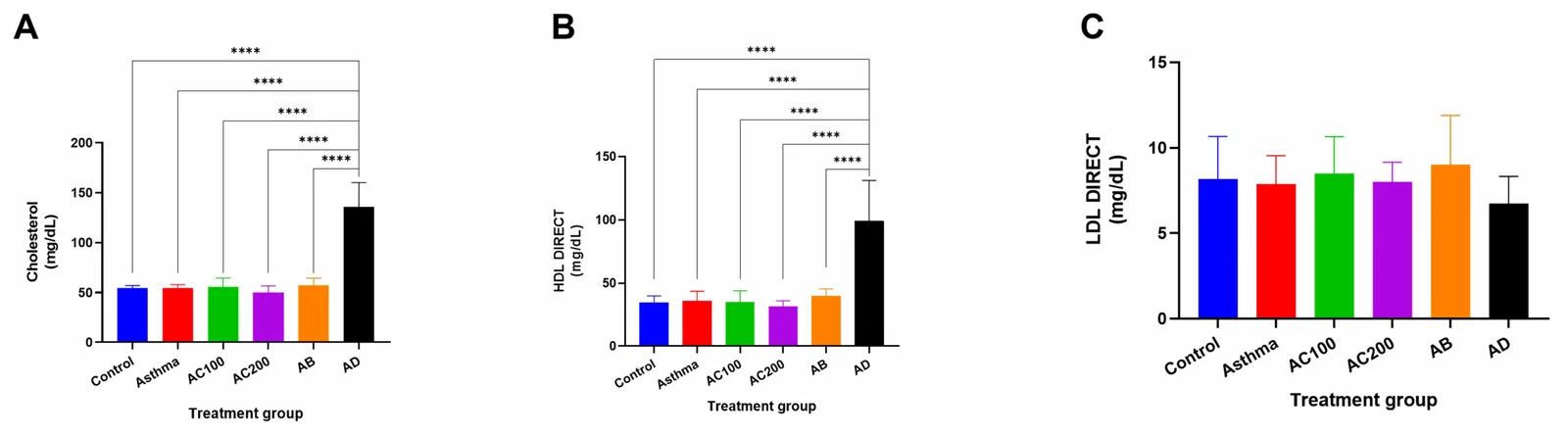
Serum lipid parameters across treatment groups. (**A**) Total Cholesterol, (**B**) HDL *(direct assay)*, and (**C**) LDL *(direct assay)*. Total cholesterol and HDL cholesterol concentrations were significantly higher in AD than in each of the other groups (**** *p* < 0.0001 for each comparison). Neither parameter differed significantly among C, A, AC100, AC200, and AB (*p* > 0.05). LDL cholesterol concentrations did not differ significantly among groups (*p* > 0.05). Values are presented as mean ± SD. Statistical comparisons were performed using one-way ANOVA followed by Tukey’s multiple-comparisons test. Asterisks denote **** *p* < 0.0001 for the indicated comparisons. C, healthy control; A, untreated asthma; AC100 and AC200, asthma treated with crocodile meat protein hydrolysate at 100 and 200 mg/kg, respectively; AB, asthma treated with chicken essence; AD, asthma treated with dexamethasone.

Serum biochemical parameters are presented in Figure 2. ALT (502.1 ± 164.8 U/L) and AST (832.1 ± 502.2 U/L) activities were significantly higher in the AD group than in all the other groups (*p* < 0.0001). Serum ALP activity was significantly lower in the AD group than the control group (*p* < 0.05), whereas albumin and total protein concentrations were significantly higher in the AD group than in all the other groups (*p* < 0.0001). ALT and AST activities in the AC100 and AC200 groups did not differ significantly from those in the control or untreated asthma group (*p* > 0.05). Serum creatinine concentrations were lower in the AD group than in all the other groups (*p* < 0.05). BUN concentrations were significantly higher in the untreated asthma, AC100, AC200, and AB groups than in the control and AD groups (*p* < 0.0001). The AD group also exhibited significantly increased total cholesterol and HDL-C concentrations compared with those in all the other groups (*p* < 0.0001; Figure 3). LDL-C concentrations did not differ significantly among the groups (*p* > 0.05). Serum lipid parameters in the AC100 and AC200 groups were comparable to those of the control and untreated asthma groups.

### 3.3. Effect of Crocodile Meat Protein Hydrolysate on Serum Antioxidant Enzyme Activities

**Figure 4:**
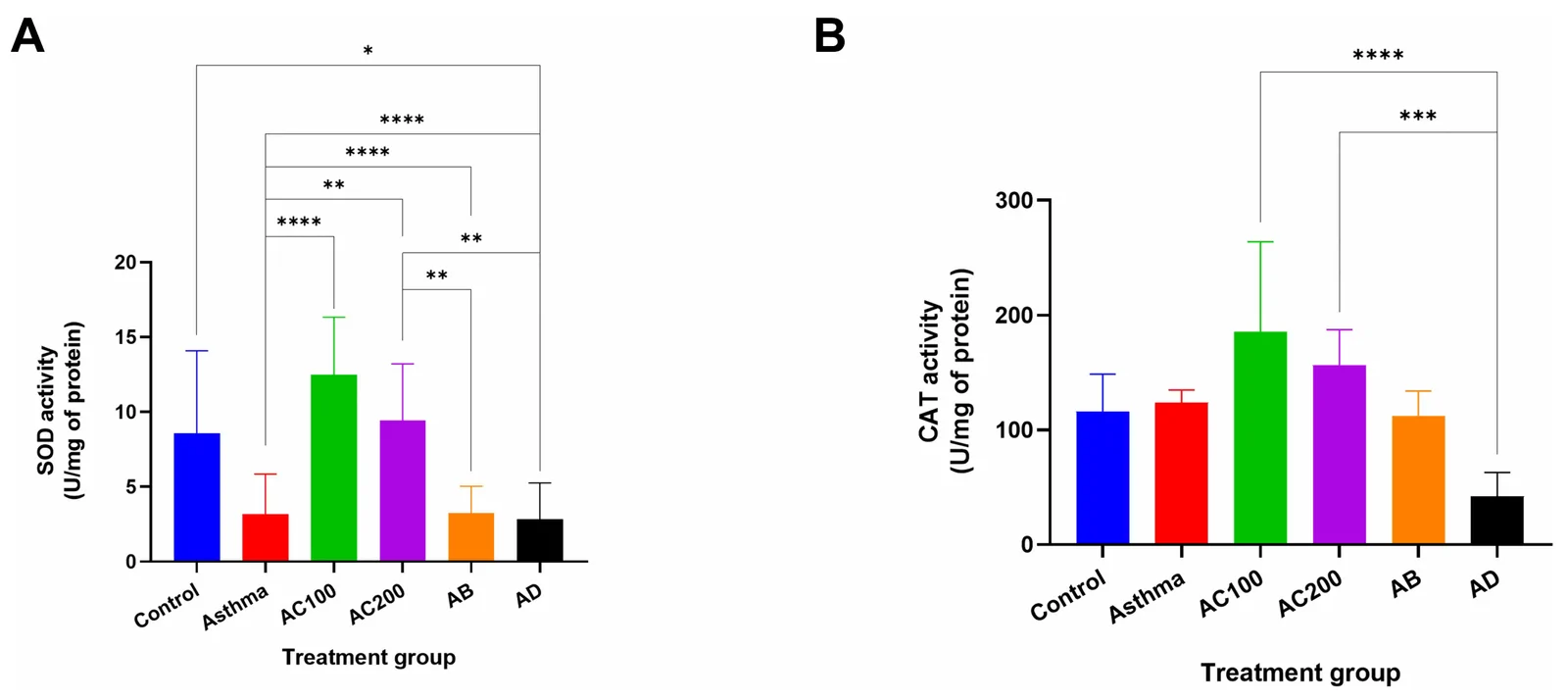
Serum SOD (**A**) and CAT (**B**) activities, expressed as U/mg protein. Values are expressed as mean ± SD. Analysis was reported as one-way ANOVA with Tukey’s multiple comparisons. Asterisks indicate statistically significant differences (* *p* < 0.05, ** *p* < 0.01, *** *p* < 0.001, **** *p* < 0.0001).

Serum antioxidant enzyme activities are presented in Figure 4. SOD activity (Figure 4A) was significantly higher in the AC100 (12.47 ± 3.87 U/mg protein) and AC200 (9.41 ± 3.80 U/mg protein) groups than in the untreated asthma (3.15 ± 2.70 U/mg protein) group (*p* < 0.0001 and *p* < 0.001, respectively). Furthermore, SOD activity in the AC100 and AC200 groups was significantly increased compared with that in the AB (3.23 ± 1.80 U/mg protein) and AD (2.84 ± 2.42 U/mg protein) groups (*p* < 0.001). While CAT activity (Figure 4B) was numerically higher in the AC100 (185.28 ± 78.63 U/mg protein) and AC200 (156.39 ± 30.88 U/mg protein) groups than in the control (115.96 ± 32.54 U/mg protein), untreated asthma (123.72 ± 11.13 U/mg protein), and AB (112.34 ± 21.57 U/mg protein) groups, these differences were not statistically significant (*p* > 0.05). In contrast, CAT activity was significantly lower in the AD group than in the AC100 and AC200 groups (*p* < 0.0001 and *p* < 0.001, respectively).

### 3.4. Effect of Crocodile Meat Protein Hydrolysate on Tracheal and Pulmonary Histopathology

**Figure 5:**
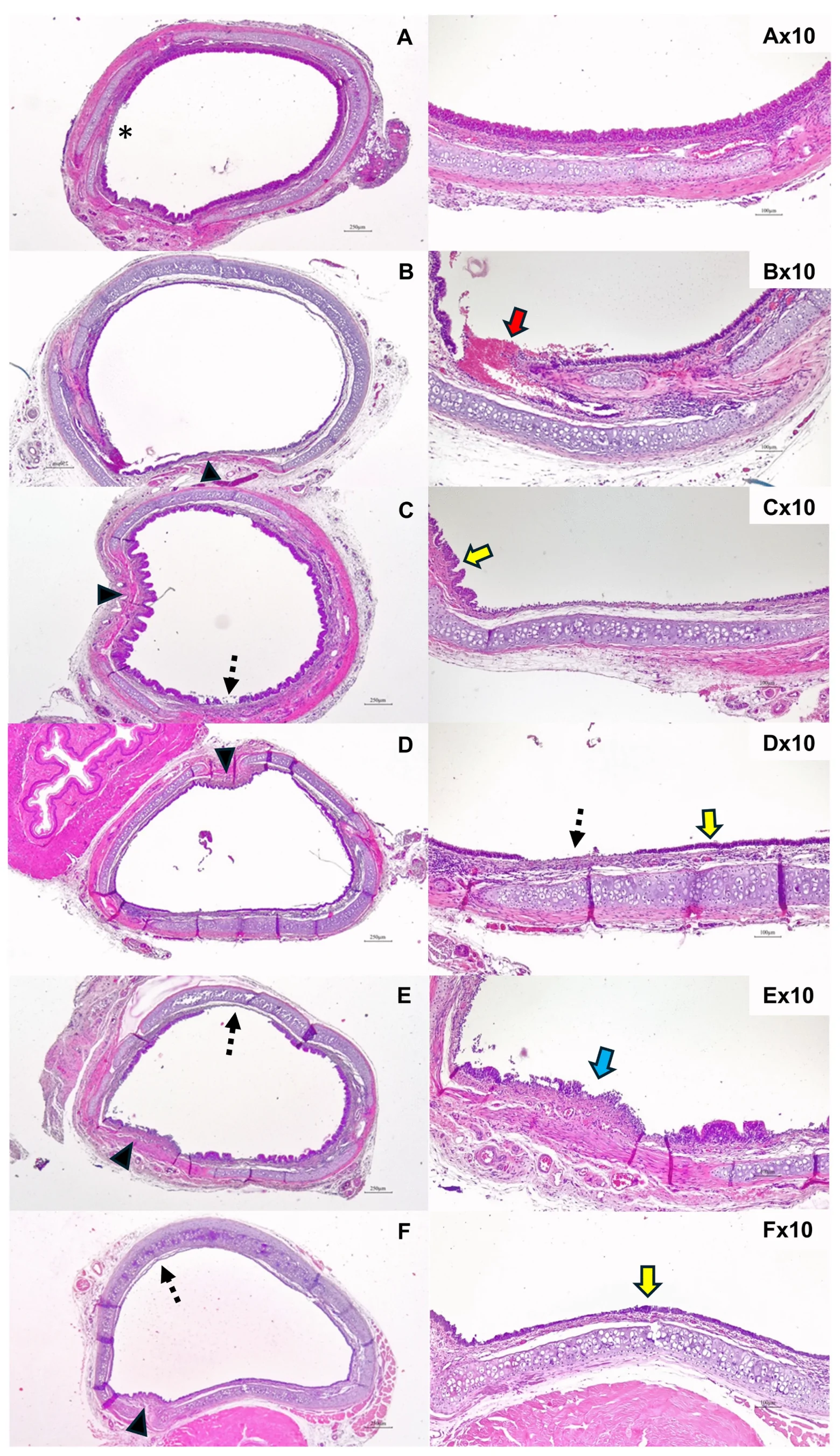
The histopathological examination of tracheal samples collected from the rat. (**A**); Control, (**B**); Asthma, (**C**); AC100, (**D**); AC200, (**E**); AB; (**F**); AD with magnifications of 4× at the left panel and 10× at the right panel. (*: indicates focal areas of epithelial cell damaged; Black arrow: indicates epithelial cell damage; red arrow: indicates tracheal mucosa hemorrhage, dash arrow: indicates epithelial desquamation; blue arrow: indicates epithelial detachment; yellow arrow: indicates cuboidal cells thinner and lacks apical cilia).

Representative H&E-stained sections are illustrated in Figure 5. The control group exhibited an intact ciliated pseudostratified columnar epithelium, with no evident submucosal inflammatory cell infiltration or luminal hemorrhage (Figure 5A). In contrast, the untreated asthma group showed moderate epithelial desquamation, loss of apical cilia, submucosal inflammatory cell infiltration, and luminal hemorrhage (Figure 5B). These findings are presented qualitatively, without quantitative lesion scoring or inferential statistical comparisons.

The representative tracheal section from AC100 and AC200 groups showed mild epithelial desquamation or ciliary loss and mild submucosal inflammatory cell infiltration remained evident. No luminal hemorrhage was evident in the displayed fields (Figure 5C,D). The AB and AD groups exhibited moderate epithelial desquamation or ciliary loss and submucosal inflammatory cell infiltration without evident luminal hemorrhage in the displayed fields (Figure 5E,F). Loss of apical cilia remained evident in the AD group. Overall descriptive histopathological suggested less pronounced tracheal lesions in the illustrated AC100 and AC200 groups than in the untreated asthma group. However, no animal-level quantitative assessment or inferential statistical comparative was performed.

**Figure 6:**
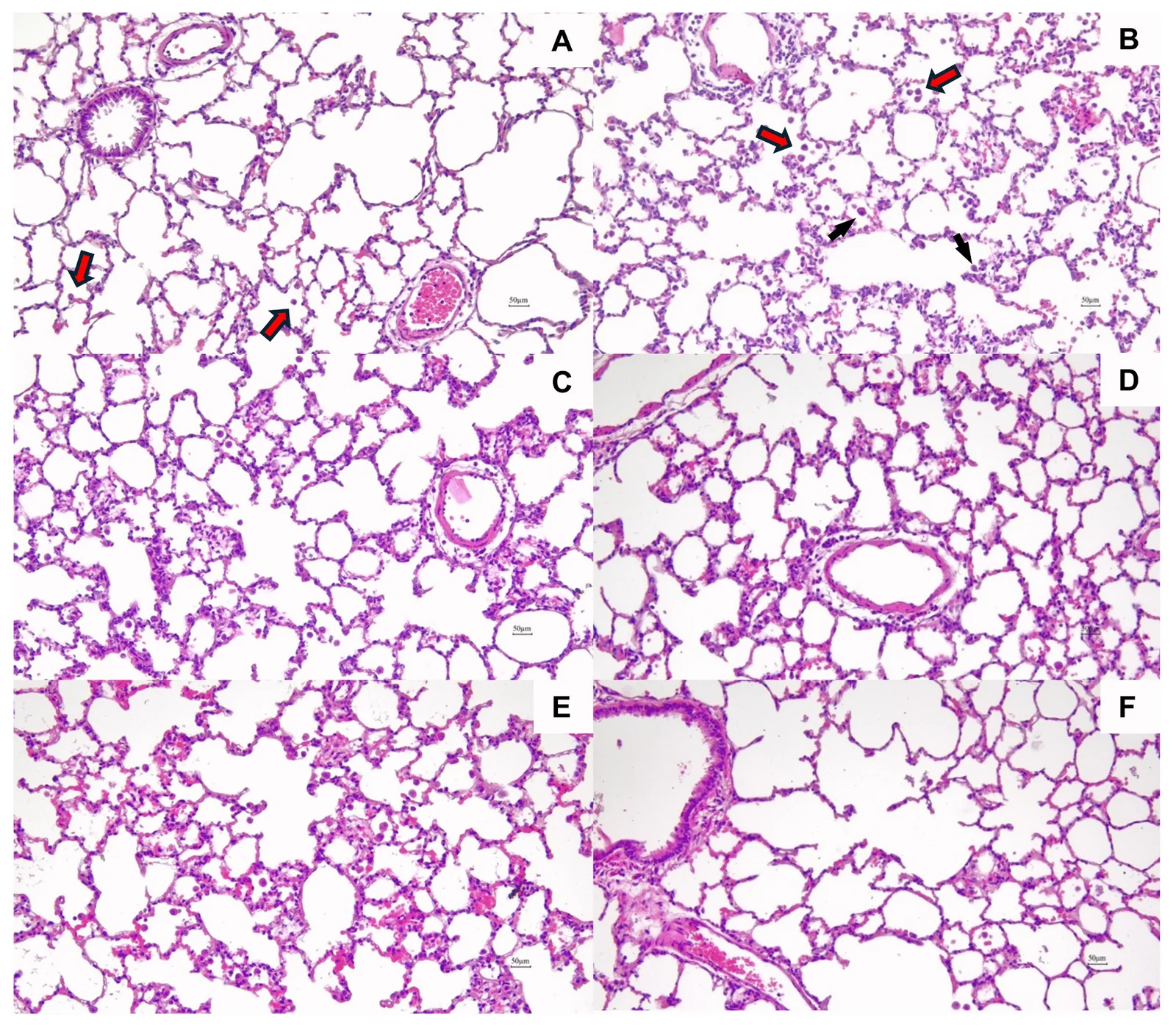
The histopathological examination of pulmonary tissue collected from the rats. (**A**); Control, (**B**); Asthma, (**C**); AC100, (**D**); AC200, (**E**); AB; (**F**); AD with 20x magnification. (Red arrow: indicates alveolus macrophages; black arrow: indicates mast cells).

Representative H&E-stained lung sections are shown in Figure 6. The control group displayed preserved pulmonary architecture, intact bronchiolar epithelium, and preserved alveolar and vascular morphology, without evident pathological inflammatory infiltration, alveolar hemorrhage, vascular congestion, or edema (Figure 6A). Occasional cells morphologically consistent with alveolar macrophages and bronchus-associated lymphoid tissue were also observed. In contrast, the untreated asthma (A) group showed prominent peribronchial and perivascular inflammatory cell infiltration, disruption of alveolar architecture with focal hemorrhage, and vascular congestion and edema (Figure 6B). Inflammatory cells were evident within the affected areas. The presentative AC100 group section showed relatively preserved alveolar architecture, with residual peribronchial or perivascular inflammatory infiltration and vascular congestion or edema. Alveolar hemorrhage was not evident in the displayed field (Figure 6C). The AC200 group showed preserved alveolar and vascular morphology, with little apparent inflammatory infiltration and no evident hemorrhage, congestion, or edema in the displayed field (Figure 6D). The AB group showed peribronchial or perivascular inflammatory infiltration, focal alveolar injury or hemorrhage, and vascular congestion or edema (Figure 6E). The AD group showed relatively preserved alveolar architecture, with little apparent inflammatory infiltration and no evident vascular congestion or edema in the displayed field (Figure 6F).

Overall, neither hydrolysate dose was associated with significant differences in body weight or most routine hematological and biochemical parameters relative to untreated asthma. Serum SOD activity was higher in both hydrolysate groups, whereas CAT activity did not differ significantly from untreated asthma. Qualitative histopathological observations suggested less pronounced tracheal and pulmonary lesions, although no animal-level quantitative assessment or statistical comparison was performed. BUN concentrations were elevated in the untreated asthma and hydrolysate groups relative to healthy controls.

## 4. Discussion

The present study showed that Siamese crocodile meat protein hydrolysate enhanced serum antioxidant enzyme activity and was associated with less pronounced tracheal and pulmonary lesions in a rat model of OVA-induced allergic asthma. Neither hydrolysate dose tested produced marked alterations in body weight or most measured hematological, hepatic, renal, and lipid parameters relative to the control or untreated asthma group, although BUN concentrations were elevated, which warrants further investigation. In contrast, dexamethasone treatment was linked to lower body weight, neutrophilia, lymphopenia, increased serum transaminase activities, and altered protein and lipid profiles. These findings illustrate the potential of Siamese crocodile meat protein hydrolysate as a food-derived bioactive ingredient and potential adjunctive intervention for allergic asthma. Additional studies incorporating airway function, inflammatory biomarkers, peptide characterization, and statistically powered histopathological analyses are required to confirm its efficacy, safety, and mechanisms of action.

Total white blood cell counts did not differ significantly among the groups, indicating that neither dose of crocodile meat protein hydrolysate produced a detectable effect on circulating leukocyte numbers. Moreover, neutrophil and lymphocyte percentages in the AC100 and AC200 groups were comparable to those of the control and untreated asthma (A) group. Conversely, dexamethasone treatment resulted in marked neutrophilia (83.7%) and lymphopenia (8.7%), a pattern characteristic of a corticosteroid-induced stress leukogram. This response may result from glucocorticoid-mediated neutrophil demargination and redistribution of circulating lymphocytes. Ultimately, peripheral leukocyte profiles alone are insufficient to establish a systemic anti-inflammatory effect. Peripheral eosinophil percentages remained low and were not significantly different among the groups. The absence of peripheral eosinophilia may reflect the timing of blood collection, redistribution of eosinophils from the circulation to airway tissues, or characteristics of the SD rat model and the sensitization protocol. Peribronchial and perivascular inflammatory infiltrates were observed histologically in untreated asthmatic rats; however, the cellular composition of these infiltrates was not confirmed using differential staining or immunohistochemistry. Consequently, eosinophilic airway inflammation could not be established through these histopathological findings alone.

This pattern of hematological modulation is consistent with observations by Cellat et al. [26], who demonstrated that tyrosol, a natural phenolic compound, significantly suppressed OVA-induced leukocytosis in Wistar rats while preserving normal leukocyte subset ratios, whereas dexamethasone treatment induced pronounced lymphopenia. Similarly, Mundo-Franco et al. [27] reported that C-phycocyanin, a natural pigment from cyanobacteria, attenuated leukocytosis and tissue myeloperoxidase activity in a Sprague-Dawley rat model of OVA-induced asthma. This achieved immune modulation without the immunosuppressive profile observed with methylprednisolone, closely paralleling the immunomodulatory benefits demonstrated by the crocodile extracts in the present study. The hematological and biochemical differences observed in the dexamethasone group require cautious interpretation. Neutrophil and lymphocyte proportions do not, by themselves, establish changes in absolute cell numbers or immune function. Likewise, differences in aminotransferase activities, albumin, and lipid parameters are insufficient to establish a specific mechanism or confirm hepatic toxicity. Additional targeted assessments would be required to determine their biological and pathological significance. These findings also do not establish that the hydrolysate is safer than dexamethasone.

The hepatoprotective profile of the crocodile extracts was one of the most compelling findings of this study. Serum ALT and AST levels in the AD group (502.1 and 832.1 U/L, respectively) exceeded control values by more than 10-fold, consistent with severe hepatocellular necrosis and potential drug-induced liver injury. The paradoxical suppression of ALP in the AD group is recognized in rodent-specific response to glucocorticoids, mediated by the downregulation of intestinal and hepatic ALP isoenzymes [4]. In contrast, AC-treated animals maintained liver enzyme profiles that were statistically indistinguishable from those of healthy controls. The present findings do not establish anti-inflammatory or hepatoprotective effects of the hydrolysate. Inflammatory cytokines, inflammatory cell activity, liver histology, and markers of oxidative damage were not assessed. Qualitatively less pronounced inflammatory infiltration cannot demonstrate suppression of inflammatory pathways, and serum biochemical findings alone do not establish hepatic protection. Similarly, higher serum SOD activity does not directly demonstrate reduced tissue oxidative damage. Anti-inflammatory and tissue-protective actions therefore remain hypotheses requiring targeted investigation rather than established explanations for the observed findings.

The extent of the dexamethasone-induced increase in transaminase activities observed in the present study is consistent with previous studies detailing hepatotoxicity induced by corticosteroid treatment in rodents. For instance, Hassan et al. [28] reported that dexamethasone administration in Wistar rats resulted in significant increases in ALT (+121%) and AST (+154%) activities relative to controls, accompanied by hyperlipidemia and oxidative stress, findings closely mirroring those noted in the AD group in the present study. Osman et al. [29] further confirmed this pattern of corticosteroid-induced hepatotoxicity, reporting that dexamethasone-treated rats exhibited alterations in key liver function markers, including transaminases and serum albumin levels, and the albumin-to-globulin ratio. In contrast, the mechanism underlying the hepatoprotective effects of the crocodile extracts is consistent with findings by Park et al. [30], who showed that a krill protein hydrolysate fraction (1–3 kDa) significantly restored hepatic SOD, CAT, and GPx activities while reducing serum transaminase levels in alcohol-treated mice. The accompanying histopathological improvements in that model directly parallel the tissue protection observed with AC treatment in the present study. These findings support the hypothesis that food-derived bioactive peptides, irrespective of species origin, share a common hepatoprotective mechanism mediated through the upregulation of endogenous antioxidant enzyme systems.

Oxidative imbalance provides a biological rationale for investigating food-derived peptides in allergic asthma, given the involvement of reactive oxygen species in airway inflammation and structural injury [31]. Previous studies of crocodile meat-derived peptides have reported antioxidant activity, although their preparation methods and experimental conditions differed from those used in the present study. Findings from crocodile blood components and hemoglobin hydrolysates concern different starting materials and cannot directly establish the properties of our meat protein hydrolysate. The active constituents of the preparation used here were not characterized; therefore, its biological effects cannot be attributed to specific peptides or peptide structure–activity relationships.

In the present study, serum SOD activity was higher in both hydrolysate-treated groups than in untreated asthma. However, CAT activity did not differ significantly from untreated asthma, although it was higher than in the dexamethasone group. These findings do not demonstrate restoration of overall antioxidant capacity or reduced oxidative damage within pulmonary tissues. Less pronounced airway lesions were also described in the hydrolysate-treated groups, but histopathological findings were qualitative and were not supported by animal-level quantitative comparisons. The occurrence of higher serum SOD activity and apparently less pronounced lesions in the same treatment groups represents descriptive co-occurrence, rather than a demonstrated animal-level correlation or causal relationship. No mediation analysis or experimental manipulation of antioxidant pathways was performed. Accordingly, antioxidant responses remain a possible explanation requiring further investigation, rather than an established mechanism of tissue protection. Direct measurements of pulmonary oxidative damage, quantitative histopathology, and experiments testing the involvement of antioxidant pathways are needed to determine whether these responses contribute to any reduction in airway injury. Phosri et al. [14] reported that blood components of *C. siamensis*, including hemoglobin, crude leukocyte extract, and plasma, exhibited notable antioxidant capacity in multiple radical scavenging assays, and additionally demonstrated anti-inflammatory activity through the reduction in nitric oxide and IL-6 production in lipopolysaccharide-stimulated RAW 264.7 macrophages. At the peptide level, Yang et al. [12] isolated four antioxidant peptides from *C. siamensis* meat hydrolysates prepared by papain digestion, including VPPHIY and RNGLPGPIGPAG, which showed half-maximal free radical scavenging activity (IC50) at 1.0 mg/mL comparable to that of ascorbic acid, with the <3 kDa fraction demonstrating the highest potency. More recently, Song et al. [13] identified eight novel antioxidant peptides from natural protease hydrolysates of *C. siamensis* myofibrillar protein through virtual screening and bioinformatics, further confirming the potential of this species as a source of antioxidant peptides. Lueangsakulthai et al. [15] demonstrated that pepsin hydrolysates of *C. siamensis* hemoglobin yielded both antioxidant and anti-inflammatory peptides. Peptide fractions isolated after 2 h of hydrolysis substantially reduced IL-6, IL-1β, and PGE2 production in lipopolysaccharide-stimulated macrophages, with active sequences identified including KIYFPHF, SAFNPHEKQ, and IIHNEKVQAHGKKVL. Together, these studies establish a clear mechanistic basis for the antioxidant and anti-inflammatory activities observed with crocodile extracts in the present study, extending previously reported in vitro bioactivities into an in vivo asthma model.

The histopathological findings corroborated the biochemical and hematological data. In tracheal and pulmonary tissues of AC-treated animals, the hallmark features of allergic airway disease—epithelial desquamation, inflammatory cell infiltration, vascular congestion, and goblet cell hyperplasia—were consistently attenuated relative to the untreated asthma group [32,33,34]. The partial preservation of ciliated epithelial integrity [35,36,37] in AC100- and AC200-treated animals is particularly noteworthy, as cilia are essential for mucociliary clearance and their loss is a critical early step in asthmatic airway remodeling. Although dexamethasone elicited profound immunosuppressive effects, it did not fully abrogate structural change in tracheal or pulmonary tissues [38,39,40]. This outcome highlights a clear limitation of standalone systemic anti-inflammatory therapeutic strategies. Notably, the crocodile extracts yielded broader, more consistent protection across both hematological and histopathological endpoints by acting through combined anti-inflammatory and tissue-protective pathways [41,42]. It is also noteworthy that while the administration of commercial chicken essence (100 mg/kg) elicited intermediate protective effects broadly comparable to those observed in the lower-dose hydrolysate group (AC100 group), increasing the hydrolysate dose to 200 mg/kg (AC200 group) yielded superior antioxidant indices and lower lesion scores compared with both the AC100 and AB groups. This outcome suggests that the specific amino acid composition and bioactive peptide profile of *C. siamensis* muscle protein hydrolysate provide greater therapeutic benefit at higher concentration than standard chicken essence. These histopathological outcomes align closely with findings reported in analogous natural product intervention studies using rodent models of OVA-induced asthma. Shakerinasab et al. [43] demonstrated that Nasturtium officinale hydroalcoholic extract significantly attenuated airway inflammatory cell infiltration, goblet cell hyperplasia, and oxidative stress markers in OVA-sensitized Wistar rats, with lung SOD and GPx activities showing substantial preservation relative to untreated model animal outcomes that closely mirror the histopathological and biochemical improvements observed in the AC groups in the present study. Similarly, Kim et al. [44] reported that a combination extract of Chinese quince and Saururus chinensis significantly reduced inflammatory cell infiltration and mucus hypersecretion in the airways of OVA-treated mice while simultaneously suppressing IL-4, IL-5, IL-13, and inducible nitric oxide synthase expression in lung tissue. These effects were mechanistically analogous to those attributed to the AC hydrolysates through the antioxidant and anti-inflammatory activities of their peptide.

The lipid and metabolic data further substantiate the favorable safety profile of the AC hydrolysates. Glucocorticoid-induced hyperlipidemia, exemplified by the 2.5-fold and near 3-fold increases in total cholesterol and HDL-C concentration in the AD group, is a well-recognized metabolic consequence of steroid therapy linked to increased cardiovascular risk [4]. Crocodile hydrolysate-treated groups maintain cholesterol homeostasis comparable to that of controls, consistent with a neutral or potentially favorable metabolic impact. BUN was elevated in untreated asthma as well as in both hydrolysate groups and the chicken essence group relative to healthy control. This pattern does not identify a hydrolysate-specific effect. Model-related change in nitrogen metabolism, differences in protein intake, catabolic processes or hydration are possible explanations, but were not established experimentally. The findings do not show that peptide supplementation was the cause. Unchanged creatinine in the hydrolysate group does not exclude renal involvement, and the absence of urine measurements and renal histology limits interpretation. The BUN elevation therefore requires further investigation before a favorable safety profile can be claimed. These metabolic perturbations detected in the AD group are consistent with the mechanistic framework described by Malkawi et al. [45], who documented that dexamethasone-treated rats exhibited hyperglycemia, altered serum lipids, and a marked reduction in total serum ALP alongside a distinctive metabolomic profile reflecting accelerated gluconeogenesis and protein catabolism. The pattern of glucocorticoid-induced dyslipidemia recorded in the present study, characterized by an increase in both total cholesterol and HDL-C levels, aligns with the bimodal lipoprotein perturbation pattern described by Reaven et al. [46], in which high-dose glucocorticoid administration produced a doubling of serum cholesterol and HDL-C concentration coincident with the activation of hepatic lipogenesis. In contrast, the metabolic neutrality of the crocodile extracts is consistent with published data on other food-derived protein hydrolysates. Nawaz et al. [47] demonstrated that dietary supplementation with fermented chicken meat protein hydrolysate, which exhibited high antioxidant activity, did not adversely affect lipid profiles, liver enzymes, or renal parameters in male Wistar rats. This finding the safety profile observed with crocodile hydrolysate in the present study, providing further evidence that food-grade animal protein hydrolysates represent metabolically safe interventional agents.

Several limitations of the present study merit acknowledgment. The OVA-induced asthma rodent model, while extensively validated, does not fully recapitulate the complex immunological and structural heterogeneity of human asthma, particularly the neutrophilic and mixed-granulocyte endotypes increasingly implicated in severe disease [48]. Furthermore, the peptide fractions were not fully characterized at the molecular level in this study. Future work employing LC-MS/MS peptidome profiling will be essential for identifying the specific bioactive sequences responsible for the observed effects and to enable structure–activity optimization. Additionally, measuring the levels of key Th2 cytokines (IL-4, IL-5, IL-13, IL-33), IgE, and airway hyperresponsiveness indices would substantially strengthen the mechanistic interpretation in subsequent studies. Similarly, the absence of bronchoalveolar lavage fluid (BALF) analysis precluded direct quantification of airway eosinophilia and inflammatory cell differentials, which represent critical endpoints for confirming local allergic airway inflammation in this model. Subsequent investigations should incorporate BALF cytology alongside Th2 cytokine and IgE profiling to provide a comprehensive immunological characterization. The study has several limitations. The hydrolysate was not characterized by peptide sequencing or comprehensive molecular-weight profiling. Airway function, BALF cell differentials, IgE, Th2 cytokines and direct oxidative-damage markers were not measured. Histopathological scores were descriptive and lacked inferential comparisons. The commercial food comparator also limits interpretation without composition matching. Gastrointestinal stability, absorption and bioavailability of the constituent peptides remain unknown. These limitations support a cautions interpretation of the observed serum SOD and histopathological finding rather than a demonstrated therapeutic intervention.

## 5. Conclusions

Siamese crocodile (*C. siamensis*) meat protein hydrolysate was associated with higher serum SOD activity in OVA-induced asthma rats, whereas CAT did not differ significantly from untreated asthma. Quantitative histopathological observation suggested less pronounced airway lesions. However, a tissue protection and causal relationship with antioxidant responses remain unconfirmed because animal-level quantitative histopathology and mechanistic evidence were unavailable. Most measured hematological and biochemical parameters showed no significant differences from untreated asthma but the elevated BUN concentrations warrant further investigation. Dexamethasone was associated with multiple hematological and biochemical alterations under the study conditions. Further studies should characterize candidate bioactive using LC-MS/MS and function validation, access bioavailability and incorporate quantitative histopathology, pulmonary oxidative and inflammatory endpoints, and airway function measurement. These findings support further preclinical investigation of the hydrolysate, while its mechanisms, efficacy and safety remain to be established.

## Figures and Tables

**Table 1:** Hematological parameters of rats across all treatment groups.

Parameter	Reference Range	Group					
		C	A	AC100	AC200	AB	AD
WBCs (×10^3^/µL)	6.6–12.6	13.2 ± 2.8	13.5 ± 1.6	11 ± 3.1	11 ± 2.5	12.5 ± 3.3	10.4 ± 7.8
Neutrophils (%)	9–35	19.4 ± 9.4 ^a^	17.3 ± 10 ^a^	33.1 ± 29.2 ^a^	27.4 ± 15.7 ^a^	26.8 ± 10.8 ^a^	83.7 ± 2.5 ^b^
Lymphocytes (%)	65–85	77.8 ± 10.6 ^a^	80 ± 11.6 ^a^	63.1 ± 33.6 ^a^	68.6 ± 13.7 ^a^	70.1 ± 10 ^a^	8.7 ± 2.4 ^b^
Monocytes (%)	0–5	2.8 ± 1.9	2.7 ± 3.2	3.5 ± 4.5	3.2 ± 3.8	2.7 ± 2.6	7.3 ± 1.5
Eosinophils (%)	0–5	0.1 ± 0.1	0 ± 0	0.2 ± 0.4	0.5 ± 0.5	0.4 ± 0.7	0.1 ± 0.1
Basophils (%)	0–1	0 ± 0.1	0 ± 0	0.1 ± 0.2	0.2 ± 0.4	0 ± 0.1	0.2 ± 0.2
RBCs (×10^6^/µL)	6.5–9.5	8.3 ± 0.7	8.5 ± 0.4	8.9 ± 0.8	8.3 ± 0.7	8.6 ± 0.5	8.7 ± 1.2
Hemoglobin (g/dL)	11.5–16.1	14.9 ± 1.3	15.7 ± 0.6	16.1 ± 1	15.3 ± 1.3	15.9 ± 1.1	16.7 ± 2.1
Hematocrit (%)	36–50	42.8 ± 3.7	44.3 ± 1.6	45.4 ± 3.2	43.4 ± 3.8	44.9 ± 3.3	46.6 ± 5.8
Platelet (×10^3^/µL)	500–1300	1129 ± 198.4 ^a^	961 ± 167.8 ^a^	936.6 ± 244.9 ^a^	935.7 ± 243.9 ^a^	981.4 ± 272.6 ^a^	617.8 ± 184.7 ^b^

Data are presented as means ± SD. Different superscript letters (^a, b^) within the same row indicate statistically significant differences among groups *(p* < 0.05). C = control; A = untreated asthma; AC100 = crocodile meat hydrolysate 100 mg/kg; AC200 = crocodile meat hydrolysate 200 mg/kg; AB = chicken essence 100 mg/kg; AD = dexamethasone 2.5 mg/kg. Reference ranges for each parameter were adopted from He et al. [23], Lillie et al. [24] and Wolford et al. [25].

## Data Availability

The original contributions presented in this study are included in the article. Further inquiries can be directed to the corresponding author.

## Abbreviations

A group	OVA-induced asthma control
AB group	OVA-induced asthma treated with commercial chicken essence at 100 mg/kg
AC100 group	OVA-induced asthma treated with crocodile meat protein hydrolysate at 100mg/kg
AC200 group	OVA-induced asthma treated with crocodile meat protein hydrolysate at 200 mg/kg
AD group	OVA-induced asthma treated with dexamethasone at 2.5 mg/kg
ALP	Alkaline phosphatase
ALT	Alanine aminotransferase
AST	Aspartate aminotransferase
BALF	Bronchoalveolar lavage fluid
BUN	Blood urea nitrogen
C group	Normal control
CAT	Serum catalase
H&E	Hematoxylin and eosin
HDL-C	High-density lipoprotein cholesterol
HPA	Hypothalamic-Pituitary-Adrenal
IACUC	Institutional Animal Care and Use Committee
IgE	Immunoglobulin E
IL	Interleukin
LDL-C	Low-density lipoprotein cholesterol
OVA	Ovalbumin
RBCs	Red blood cells
SD	Sprague-Dawley
SOD	Superoxide dismutase
Th2	T-helper 2
WBCs	White blood cells
